# Supplementary material for: Multiple targeted grassland restoration interventions enhance ecosystem service multifunctionality
Source: Nat Commun. 2025 Apr 28;16:3971. doi: 10.1038/s41467-025-59157-8 (PMC12037718; doi:10.1038/s41467-025-59157-8)
Supplement: Supplementary file 1 — Supplementary Information [file 41467_2025_59157_MOESM1_ESM.pdf]

Supplementary information for

Multiple targeted grassland restoration interventions enhance ecosystem service multifunctionality

Author list: Shangshi Liu[1,2,3*], Susan E. Ward[4], Andrew Wilby[4], Peter Manning[5], Mengyi Gong[6], Jessica Davies[4], Rebecca Killick[6], John N. Quinton[4], Richard D. Bardgett[1,4]

[1] Department of Earth and Environmental Sciences, The University of Manchester, Manchester, UK

[2] Yale School of the Environment, Yale University, New Haven, USA

[3] Yale Center for Natural Carbon Capture, Yale University, New Haven, USA

[4] Lancaster Environment Centre, Lancaster University, Lancaster, UK

[5] Department of Biological Sciences, University of Bergen, Bergen, Norway

[6] School of Mathematical Sciences, Lancaster University, Lancaster, UK

*Corresponding author: liushangshi@gmail.com or shangshi.liu@yale.edu

Table S1. Ecosystem services and associated indicators of grasslands measured in this study.

Ecosystem services	Indicators	Rationale
Forage production	Yield	Quantity of forage production.
	Quality-adjusted yield	Quality-adjusted yield quantified by the crude protein production, which is associated with revenues.
Carbon stocks and sequestration	Aboveground biomass carbon	With a mean residence time of <1 year in grazed grasslands.
	Root carbon stock	With a mean residence time of ~1 year. Also related to maintenance of soil physical stability.
	Litter carbon stock	With a mean residence time of <1 year in grazed grasslands.
	Particulate organic carbon	Mainly derived from plants, with a mean residence time of <10 years to decades.
	Mineral-associated organic carbon	Microbial or plant origin low molecular weight compounds, with a mean residence time from decades to centuries.
	Dissolved organic carbon	Active carbon pool.
	Microbial biomass	Active carbon pool with a mean resident time ranging from days to months, positively correlated with soil carbon content in grassland ecosystems.
	Net ecosystem exchange	Direct measurement of the size of C sink/source during sampling time.
Plant diversity conservation value	Plant species richness	Direct measurements of plant diversity
	Plant diversity (Simpson's index)	
Pollination	Bombus spp visitation	Direct measurement of the visitation of main taxa of pollinators visitation during sampling time.
	Syrphidade visitation	
	Diptera visitation	
Maintenance of soil nutrients	Soil nitrogen content	Direct measurement of soil nitrogen content
	Microbial nitrogen	Related to N cycling and retention of soil N.
	Microbial phosphrous	Related to P cycling and retention of soil P.
	Fungal PLFA	Related to organic matter decomposition.
	Bacterial PLFA	Related to organic matter decomposition.
Maintenance of soil physical stability	Water holding capacity	Related to soil capacity to intercept or store water resource, also related to regulation of water quality.
	Soil aggregation stability	Related to soil stability to resist water erosion
Regulation of water quality	Nitrogen leaching	Standard scores were minus by 1, so a positive value related to ability to prevent N and P leaching from ecosystem.
	Phosphorus leaching	
Aesthetic value	Flower abundance	Direct measurement of diversity and abundance of flowers, both of which are positively associated with aesthetic value.
	Flower diversity	

Table S2. Number of interventions effects on the ecosystems service multifunctionality using different modelling approaches to reveal the contributions of intervention identities and intervention interactions. AIC: Akaike information criterion, logLik: log-likelihood. Statistical analyses were performed using linear mixed-effects models (n= 48 plots), and *P* values were obtained from two-sided statistical test.

	**Coefficient**	**F value**	***P* value**	**AIC**	**logLik**
Equal weighted averaging ecosystem service multifunctionality					
Baseline model	0.05	23.48	$\mathbf{2.59\times10^{-4}}$	-127.88	63.94
Intervention identities	0.05	26.67	$\mathbf{6.02\times10^{-6}}$	-139.16	69.58
Intervention interactions	0.04	20.69	$\mathbf{1.46\times10^{-2}}$	-139.56	69.78
Regenerative agriculture prioritized ecosystem service multifunctionality					
Baseline model	0.09	14.91	$\mathbf{1.73\times10^{-3}}$	-90.05	45.03
Intervention identities	0.05	9.92	$\mathbf{3.09\times10^{-3}}$	-109.05	54.52
Intervention interactions	0.05	10.05	$\mathbf{2.99\times10^{-3}}$	-109.16	54.58
Nature conservation prioritized ecosystem service multifunctionality					
Baseline model	0.03	11.06	$\mathbf{4.99\times10^{-3}}$	-118.04	59.02
Intervention identities	0.05	26.64	$\mathbf{5.65\times10^{-6}}$	-125.16	62.58
Intervention interactions	0.05	24.22	$\mathbf{1.93\times10^{-3}}$	-125.20	62.60
Climate mitigation prioritized ecosystem service multifunctionality					
Baseline model	0.05	15.88	$\mathbf{1.35\times10^{-3}}$	-124.14	62.07
Intervention identities	0.02	5.15	$\mathbf{5.32\times10^{-2}}$	-140.00	70.00
Intervention interactions	0.02	5.25	$\mathbf{5.08\times10^{-2}}$	-140.31	70.16
Aesthetic value prioritized ecosystem service multifunctionality					
Baseline model	0.07	21.22	$\mathbf{4.08\times10^{-4}}$	-75.40	37.70
Intervention identities	0.08	27.00	$\mathbf{1.53\times10^{-4}}$	-81.44	40.72
Intervention interactions	0.08	26.59	$\mathbf{1.08\times10^{-3}}$	-81.51	40.76

Table S3. Statistical results using linear mixed-effects model for the effects of number of interventions on standardised value of individual ecosystem service indicators. *P*-values were obtained from a two-sided statistical test. Effect sizes were estimated using the coefficients of linear mixed-effects model (n= 48 plots).

	**F value**	***P* value**	**Effect size**
Yield	12.29	**0.003**	0.14
Quality adjusted yield	14.26	**0.002**	0.14
Aboveground biomass carbon	23.80	$\mathbf{2.4\times10^{-4}}$	0.15
Litter carbon stock	0.01	0.906	0.00
Root carbon stock	3.95	0.053	0.06
Dissolved organic carbon	1.16	0.300	0.03
Particulate organic carbon	5.22	**0.038**	0.09
Mineral-associated organic carbon	1.05	0.324	-0.03
Microbial biomass	1.92	0.188	0.05
Net ecosystem change	1.21	0.290	0.04
Plant diversity (Simpson's index)	0.00	0.956	0.00
Plant species richness	0.82	0.380	0.03
Diptera visitation	15.93	$\mathbf{2.4\times10^{-4}}$	0.12
Syrphidae visitation	0.00	1.000	0.00
Bombus visitation	6.66	**0.022**	0.11
Soil nitrogen content	4.27	0.058	0.06
Fungal phospholipid fatty acids	3.41	0.071	0.05
Bacterial phospholipid fatty acids	3.13	0.084	0.05
Microbial nitrogen	1.93	0.186	0.04
Microbial phosphorus	4.84	**0.045**	0.10
Soil aggregation stability	2.55	0.133	-0.06
Water holding capacity	0.43	0.516	0.02
Nitrogen leaching	0.24	0.633	-0.01
Phosphorus leaching	0.21	0.650	-0.01
Flower abundance	7.04	**0.011**	0.08
Flower diversity (Shannon index)	2.67	0.109	0.05

Table S4. Required sample sizes for different effect sizes (percentage change to the mean), given a statistical power of 0.8. These calculations are based on the means and variances of the ecosystem service indicators and multifunctionality indices. The relationship between effect size and sample size, with a statistical power of 0.8 for all ecosystem service indicators and multifunctionality indices, was illustrated using a t-test[1]. The analysis was conducted using the "pwr" package[2] in R. Required sample sizes smaller than 24 are in bold.

	**Change 20%**	**Change 30%**	**Change 40%**	**Change 50%**
Yield	53	24	**14**	**10**
Quality adjusted yield	71	32	**19**	**13**
Aboveground biomass carbon	70	32	**19**	**12**
Litter carbon stock	69	32	**18**	**12**
Root carbon stock	51	24	**14**	**9**
Dissolved organic carbon	68	31	**18**	**12**
Particulate organic carbon	33	**15**	**9**	**7**
Mineral-associated organic carbon	101	46	26	**17**
Microbial biomass	50	**23**	**14**	**9**
Net ecosystem change	30	**14**	**9**	**6**
Plant diversity (Simpson's index)	56	25	**15**	**10**
Plant species richness	40	**19**	**11**	**8**
Diptera visitation	54	25	**15**	**10**
Syrphidae visitation	69	31	**18**	**12**
Bombus visitation	149	67	38	25
Soil nitrogen content	**24**	**12**	**7**	**5**
Fungal phospholipid fatty acids	26	**12**	**8**	**6**
Bacterial phospholipid fatty acids	61	28	**16**	**11**
Microbial nitrogen	69	32	**18**	**12**
Microbial phosphorus	54	25	**15**	**10**
Soil aggregation stability	**15**	**8**	**5**	**4**
Water holding capacity	**18**	**9**	**6**	**4**
Nitrogen leaching	**10**	**5**	**4**	**3**
Phosphorus leaching	**7**	**4**	**3**	**3**
Flower abundance	42	**19**	**12**	**8**
Flower diversity (Shannon index)	**21**	**10**	**6**	**5**
Equal weighted averaging ecosystem service multifunctionality	**5**	**3**	**3**	**2**
Regenerative agriculture prioritized ecosystem service multifunctionality	**13**	**7**	**5**	**4**
Nature conservation prioritized ecosystem service multifunctionality	**6**	**4**	**3**	**3**
Climate mitigation prioritized ecosystem service multifunctionality	**6**	**4**	**3**	**3**
Aesthetic value prioritized ecosystem service multifunctionality	**8**	**5**	**3**	**3**

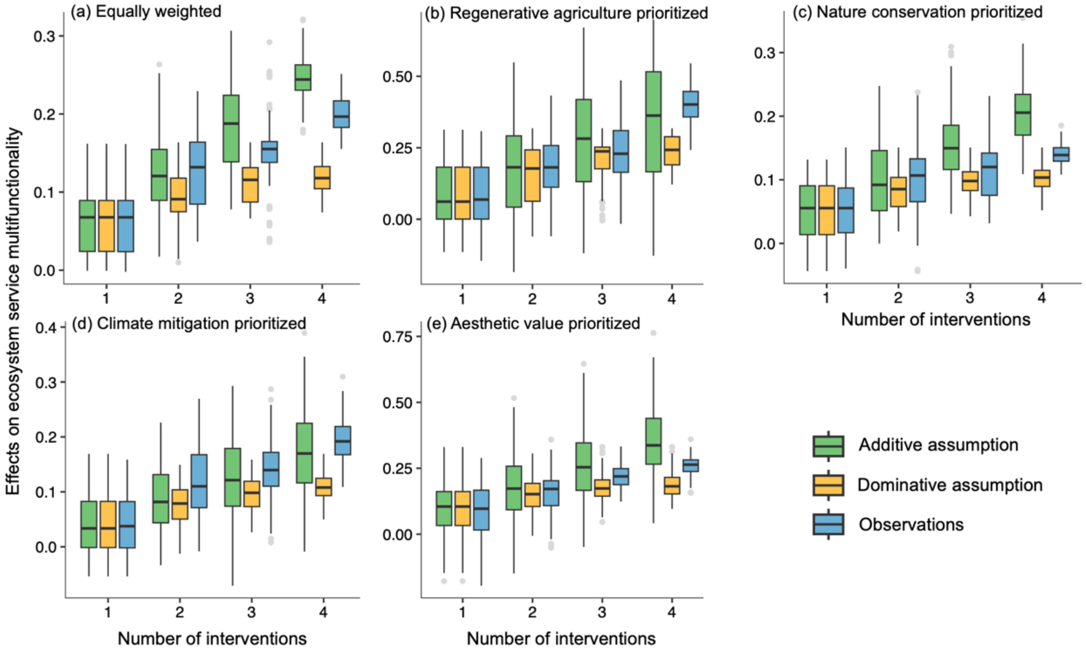

Figure S1. Effect of the number of interventions on ecosystem service multifunctionality. For each ecosystem service multifunctionality index, the effects of single interventions were estimated and then used to predict the multiple interventions effect based on either an additive or dominative assumption. The boxplots show the median (horizontal line), interquartile range (box), and whiskers extending to 1.5 times the interquartile range.

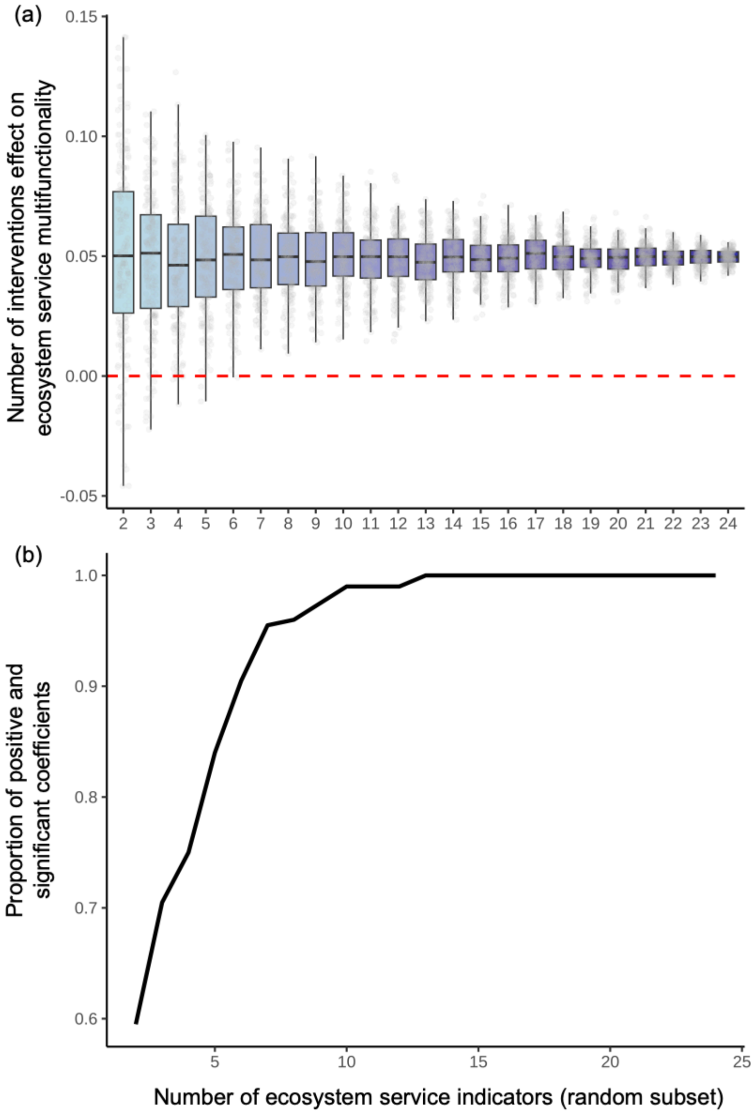

Figure S2. The effect of the number of ecosystem service indicators on the relationship between number of interventions and multifunctionality (a, b). In panel (a), Each circle represents the slope between the number of interventions and multifunctionality for a specific random subset of the 26 ecosystem service indicators measured in this study. The boxplots present the statistical results obtained from 200 random samplings conducted for different numbers of ecosystem services. The boxplots show the median (horizontal line), interquartile range (box), and whiskers extending to 1.5 times the interquartile range.

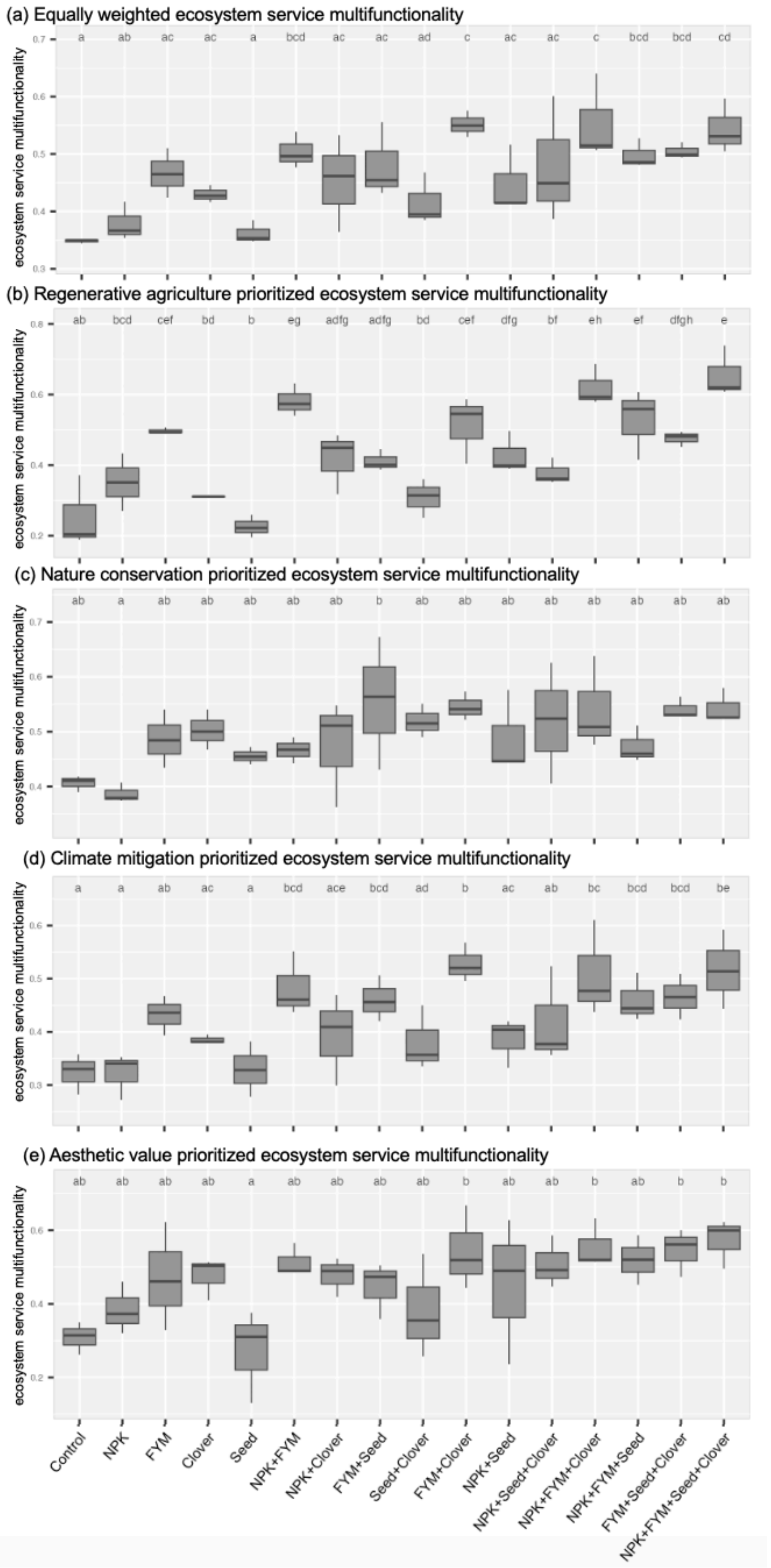

Figure S3. Effect of different combinations of interventions on ecosystem service multifunctionality. Means not sharing any letters are significant different by Tukey-test at the 5% level of significance. The boxplots show the median (horizontal line), interquartile range (box), and whiskers extending to 1.5 times the interquartile range.

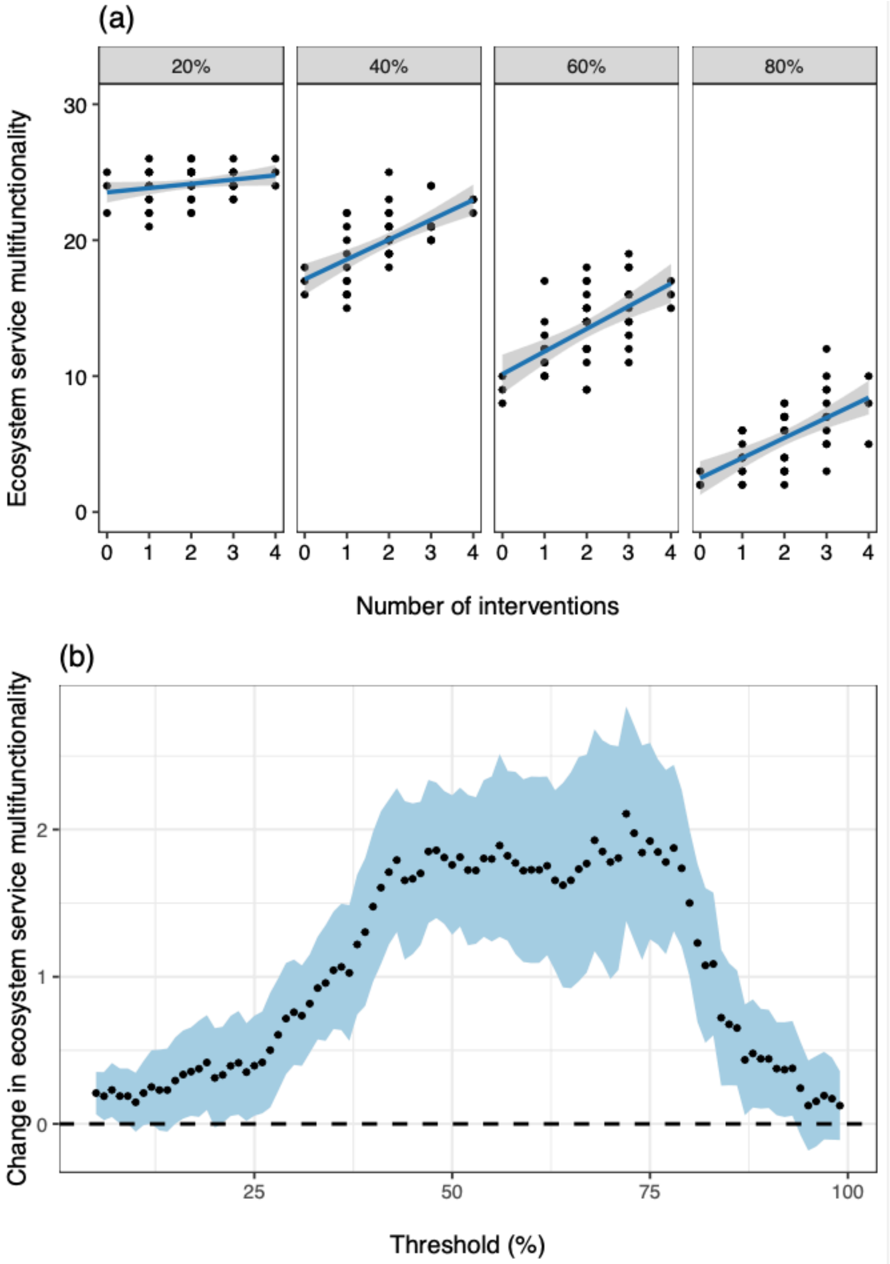

Figure S4. Effect of the number of interventions on ecosystem service multifunctionality, which is defined by the number of ecosystem service indicators that reach a specified threshold percentage of the maximum observed service indicators. The panels display the relationships of ecosystem service multifunctionality for: (a) four different thresholds (20%, 40%, 60%, and 80% of the maximum), and (b) a threshold range from 1% to 99%, as affected by the number of interventions. In panel (b) this effect is measured by the change in ecosystem service multifunctionality per additional intervention.

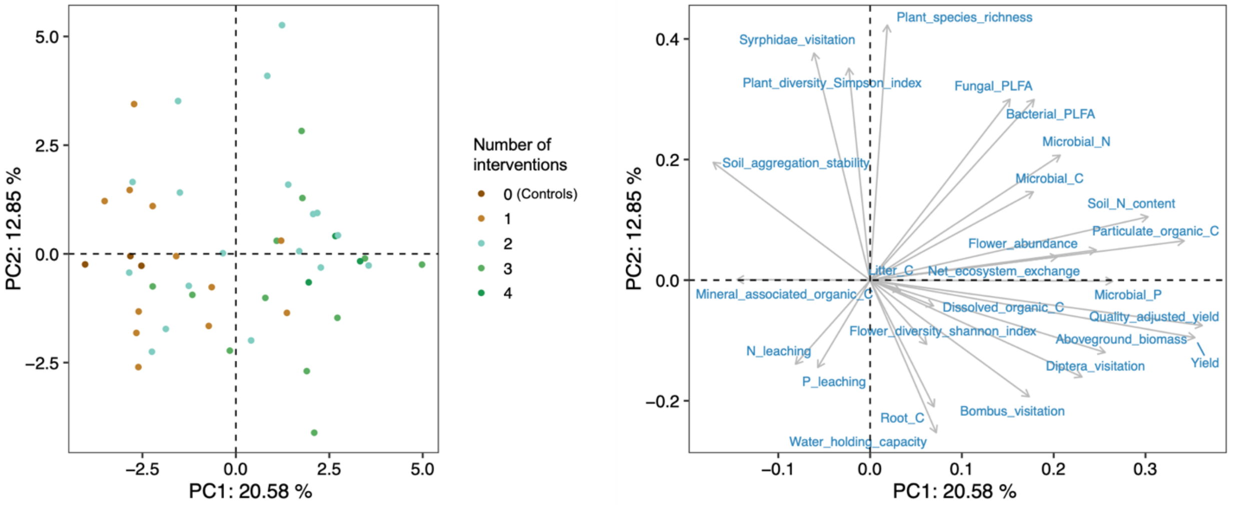

Figure S5. Principal component analysis of multiple ecosystem service indicators in this study. PLFA: phospholipid fatty acids. Only 33.43% of the variance is explained by the first two components, suggests that the dataset is highly complex and implies that ecosystem service indicators are generally not strongly correlated.

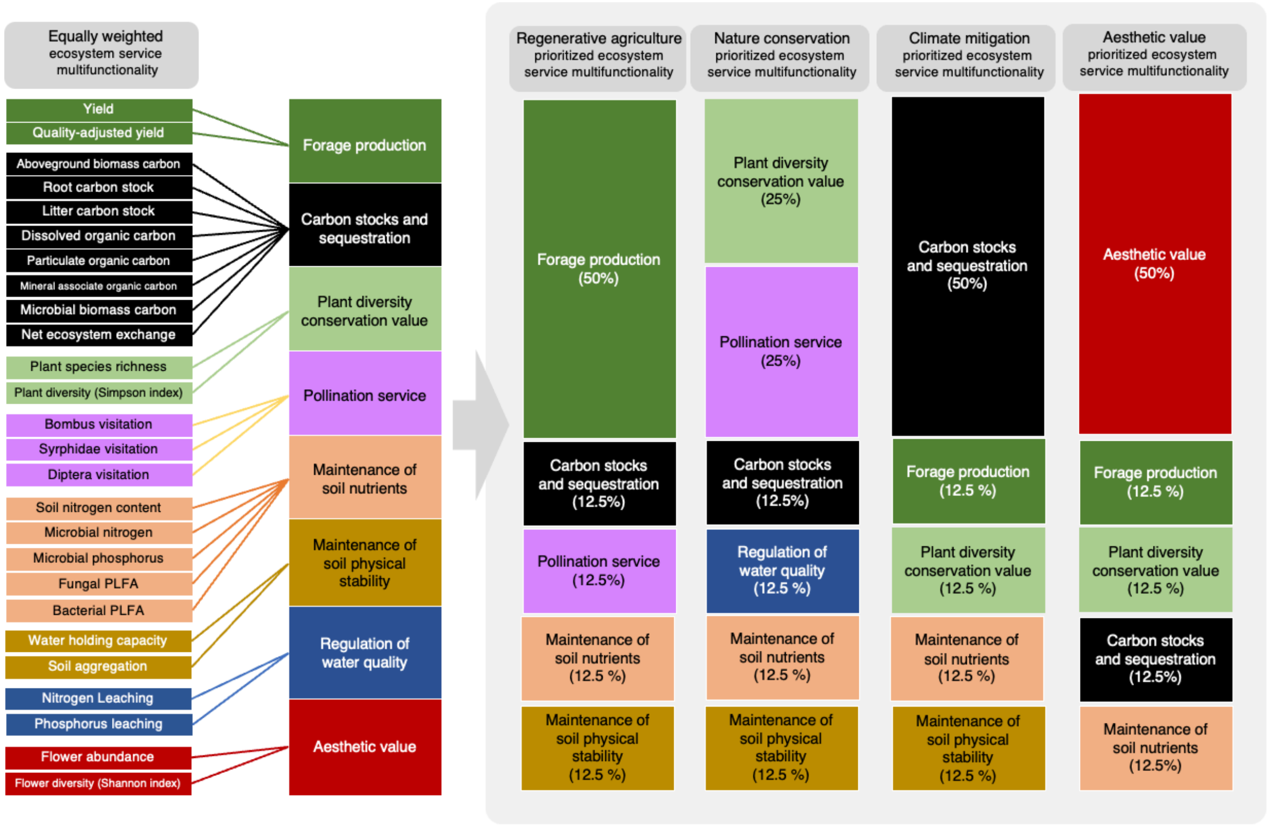

Figure S6: Ecosystem service weightings used to produce five measures of ecosystem multifunctionality according to scenarios representing different management objectives.

References:

1. Harrer, M., Cuijpers, P., Furukawa, T.A., & Ebert, D.D. (2021). *Doing Meta-Analysis with R: A Hands-On Guide*. Boca Raton, FL and London: Chapman & Hall/CRC Press. ISBN 978-0-367-61007-4
2. Champely, S. (2020). pwr: Basic Functions for Power Analysis. R package version 1.3-0, https://CRAN.R-project.org/package=pwr.
