# Supplementary material for: Multiple targeted grassland restoration interventions enhance ecosystem service multifunctionality
Source: Nat Commun. 2025 Apr 28;16:3971. doi: 10.1038/s41467-025-59157-8 (PMC12037718; doi:10.1038/s41467-025-59157-8)
Supplement: Supplementary file 2 — Reporting Summary [file 41467_2025_59157_MOESM2_ESM.pdf]

nature portfolio

Corresponding author(s): Shangshi Liu

Last updated by author(s): Feb 10, 2025

Reporting Summary

Nature Portfolio wishes to improve the reproducibility of the work that we publish. This form provides structure for consistency and transparency in reporting. For further information on Nature Portfolio policies, see our Editorial Policies and the Editorial Policy Checklist.

Statistics

For all statistical analyses, confirm that the following items are present in the figure legend, table legend, main text, or Methods section.

n/a	Confirmed	
☐	☒	The exact sample size (*n*) for each experimental group/condition, given as a discrete number and unit of measurement
☐	☒	A statement on whether measurements were taken from distinct samples or whether the same sample was measured repeatedly
☐	☒	The statistical test(s) used AND whether they are one- or two-sided<br>*Only common tests should be described solely by name; describe more complex techniques in the Methods section.*
☐	☒	A description of all covariates tested
☐	☒	A description of any assumptions or corrections, such as tests of normality and adjustment for multiple comparisons
☐	☒	A full description of the statistical parameters including central tendency (e.g. means) or other basic estimates (e.g. regression coefficient) AND variation (e.g. standard deviation) or associated estimates of uncertainty (e.g. confidence intervals)
☐	☒	For null hypothesis testing, the test statistic (e.g. *F*, *t*, *r*) with confidence intervals, effect sizes, degrees of freedom and *P* value noted<br>*Give P values as exact values whenever suitable.*
☒	☐	For Bayesian analysis, information on the choice of priors and Markov chain Monte Carlo settings
☒	☐	For hierarchical and complex designs, identification of the appropriate level for tests and full reporting of outcomes
☐	☒	Estimates of effect sizes (e.g. Cohen's *d*, Pearson's *r*), indicating how they were calculated

Our web collection on statistics for biologists contains articles on many of the points above.

Software and code

Policy information about availability of computer code

Data collection	No software was used for data collection
Data analysis	All analyses were done in R (2022), version 4.2., using the “lme4” package for mixed effect modelling and visualised with the “ggplot2” package

For manuscripts utilizing custom algorithms or software that are central to the research but not yet described in published literature, software must be made available to editors and reviewers. We strongly encourage code deposition in a community repository (e.g. GitHub). See the Nature Portfolio guidelines for submitting code & software for further information.

Data

Policy information about availability of data

All manuscripts must include a data availability statement. This statement should provide the following information, where applicable:

- Accession codes, unique identifiers, or web links for publicly available datasets
- A description of any restrictions on data availability
- For clinical datasets or third party data, please ensure that the statement adheres to our policy

All ecosystem service multifunctionality data generated in this study have been deposited in the Figshare (https://doi.org/10.6084/m9.figshare.28440020.v1).

Research involving human participants, their data, or biological material

Policy information about studies with human participants or human data. See also policy information about sex, gender (identity/presentation), and sexual orientation and race, ethnicity and racism.

Reporting on sex and gender	N/A
Reporting on race, ethnicity, or other socially relevant groupings	N/A
Population characteristics	N/A
Recruitment	N/A
Ethics oversight	N/A

Note that full information on the approval of the study protocol must also be provided in the manuscript.

Field-specific reporting

Please select the one below that is the best fit for your research. If you are not sure, read the appropriate sections before making your selection.

☐ Life sciences ☐ Behavioural & social sciences ☒ Ecological, evolutionary & environmental sciences

For a reference copy of the document with all sections, see nature.com/documents/nr-reporting-summary-flat.pdf

Ecological, evolutionary & environmental sciences study design

All studies must disclose on these points even when the disclosure is negative.

Study description

We assessed how increasing the number of restoration treatments affects ecosystem service multifunctionality, based on 26 ecosystem service indicators measured over 4 years. This study is based on a long-term grassland management experiment established in 1989, which provides a range of management-relevant experimental restoration treatments at a field scale. This restoration experiment included four treatments with their respective controls, namely:
1. Low amount of inorganic fertilizer addition (nitrogen: phosphorus: potassium = 20:10:10) at 25 kg nitrogen ha-1 y-1.
2. Farmyard manure addition at 12 t ha-1 y-1.
3. Addition of both commercial and locally sourced mixed seeds of 19 species to increase plant diversity.
4. Promotion of the N-fixing leguminous herb Trifolium pratense (red clover) by seed addition, which has been shown to increase soil nitrogen and soil carbon sequestration.
The inorganic fertilizer and mixed seeds addition treatments were established in 1990, the farmyard manure addition treatment in 1998, and the clover seed addition treatment was added in 2004 and repeated in 2011. Combined in a fully factorial design, this resulted in 16 different restoration treatment plots, each repeated in 3 blocks in a split-plot design, giving a total of 48 sampling plots. This design provides a gradient of the number of restoration interventions, ranging from 0 (i.e., control) to a maximum of 4 interventions, along with all possible combinations, allowing for the exploration of the effect of the number of interventions on ecosystem service multifunctionality.

Research sample

We quantified ecosystem service indicators using:
1. Plant shoot and root samples.
2. Soil samples to quantify carbon storage, PLFA for microbial community, soil fertility, water holding capacity, and aggregate stability.
3. Gas samples to represent net ecosystem exchange and indicate carbon sequestration.
4. Field estimation of pollinator visitation.
5. Field counting of flower abundance of each species.

Sampling strategy

We measured all ecosystem service indicators of each treatment across three blocks. The sample size of replicates wasn’t predetermined. For those ecosystem service indicators greatly influenced by differences in biotic and abiotic conditions between seasons and years, such as forage production, net ecosystem exchange, and flower abundance and diversity, we conducted multiple measurements in the field.

Data collection

Data from instruments were stored as spreadsheet files as soon as the analyses were performed. Field notes on species composition, pollinator visitation, and flower abundance were also stored as spreadsheets for further analysis.
Data collection of ecosystem service indicators was led by Susan E. Ward and Andrew Wilby, with help from Helen Quirk, Catherine Baxendale, Mike Whitfield, Gareth McShane, and Phil Donkersley. Tanya St Pierre collected the pollinator observation data, and Judith Allinson provided the vegetation survey data.

Timing and spatial scale

Fieldwork was carried out between 2011 to 2014, with each plot being 3 × 3 m. Specific measurements included:

• Forage production: Measured annually by sampling aboveground shoot tissue from a 2 × 1 m area within each treatment plot in July or August from 2011 to 2014.
• Aboveground biomass, root biomass, and litter carbon stock: Measured in summer 2013.
• Soil carbon stocks, total nitrogen content, soil microbial biomass element content, phospholipid fatty acid (PLFA) content, aggregation stability: Sampled in summer 2013.
• Net ecosystem exchange of CO2: Measured from July 2011 to August 2014 over the midday period, at monthly intervals during the growing season (May-Sept), and bi-monthly from Oct-April. Measurements of CO2 exchange were made over 120-second intervals.
• Vascular plant species survey: Carried out in each plot in summer 2014.
• Insect visitations to flowering plants: Observed in June 2014 when grazing animals were excluded from plots. Observations were carried out between 10:00 am and 3:00 pm where possible on warm sunny days (12˚C or higher < 30% cloud cover or >15˚C when overcast) without rain or strong winds (>10 mph).
• Soil nutrient retention capacity: Measured in the laboratory in 2013, using intact soil cores from the field site.
• Flower abundance and diversity: Observed eight times during the peak flowering period (mid-June to mid-July) of 2014.

Data exclusions	No data was excluded.
Reproducibility	All instruments were checked for accuracy and reproducibility. As this is a real-world field experiment, for those ecosystem service indicators greatly influenced by differences in biotic and abiotic conditions between seasons and years, such as forage production, net ecosystem exchange, and flower abundance and diversity, we conducted multiple measurements in the field.
Randomization	This experiment used a split-plot design. Within each main plot, the subplot treatments were randomly assigned. This ensures that each level of the subplot factor is randomly distributed within each main plot.
Blinding	Full blinding was not possible in the field study, as the treatments created obvious appearances of ecosystems (e.g., flower abundance). However, we processed all samples in the lab with randomly allocated numbers rather than labeled by the treatments.

Did the study involve field work? ☒ Yes ☐ No

Field work, collection and transport

Field conditions	The soil is a shallow brown-earth of moderate-high residual fertility over limestone. The plant community, when the experiment was established, was described as agriculturally improved, plant species-poor grassland dominated by Lolium perenne and Cynosurus cristatus. The mean annual temperature is 7.3˚C, and mean annual precipitation is ~1400 mm.
Location	Colt Park Meadows, Ingleborough National Nature Reserve in northern England, United Kingdom<br>Latitude 54°12’N, Longitude 2°21’W, 350 m.a.s.l.
Access & import/export	We have obtained support from Natural England, the landowner, for this long-term experiment. No samples needed to be imported or exported.
Disturbance	This study did not pose major disturbances to the grassland ecosystem.

Reporting for specific materials, systems and methods

We require information from authors about some types of materials, experimental systems and methods used in many studies. Here, indicate whether each material, system or method listed is relevant to your study. If you are not sure if a list item applies to your research, read the appropriate section before selecting a response.

Materials & experimental systems

n/a	Involved in the study	
☒	☐	Antibodies
☒	☐	Eukaryotic cell lines
☒	☐	Palaeontology and archaeology
☒	☐	Animals and other organisms
☒	☐	Clinical data
☒	☐	Dual use research of concern
☐	☒	Plants

Methods

n/a	Involved in the study	
☒	☐	ChIP-seq
☒	☐	Flow cytometry
☒	☐	MRI-based neuroimaging

Dual use research of concern

Policy information about [dual use research of concern]

Hazards

Could the accidental, deliberate or reckless misuse of agents or technologies generated in the work, or the application of information presented in the manuscript, pose a threat to:

No	Yes	
☒	☐	Public health
☒	☐	National security
☒	☐	Crops and/or livestock
☒	☐	Ecosystems
☒	☐	Any other significant area

Experiments of concern

Does the work involve any of these experiments of concern:

No	Yes	
☒	☐	Demonstrate how to render a vaccine ineffective
☒	☐	Confer resistance to therapeutically useful antibiotics or antiviral agents
☒	☐	Enhance the virulence of a pathogen or render a nonpathogen virulent
☒	☐	Increase transmissibility of a pathogen
☒	☐	Alter the host range of a pathogen
☒	☐	Enable evasion of diagnostic/detection modalities
☒	☐	Enable the weaponization of a biological agent or toxin
☒	☐	Any other potentially harmful combination of experiments and agents

Plants

Seed stocks	N/A
Novel plant genotypes	N/A
Authentication	N/A
